# The Inhibition of Spinal Astrocytic JAK2-STAT3 Pathway Activation Correlates with the Analgesic Effects of Triptolide in the Rat Neuropathic Pain Model

**DOI:** 10.1155/2012/185167

**Published:** 2012-12-29

**Authors:** Jun Tang, Zhi-Hong Li, Shun-Nan Ge, Wei Wang, Xiao-Peng Mei, Wen Wang, Ting Zhang, Li-Xian Xu, Jin-Lian Li

**Affiliations:** ^1^Department of Anatomy, Histology and Embryology and K. K. Leung Brain Research Centre, The Fourth Military Medical University, Xi'an 710032, China; ^2^Department of Anesthesiology, School of Stomatology, The Fourth Military Medical University, Xi'an 710032, China; ^3^Department of Neurosurgery, Tangdu Hospital, The Fourth Military Medical University, Xi'an 710038, China

## Abstract

Neuropathic pain (NP) is an intractable clinical problem without satisfactory treatments. However, certain natural products have been revealed as effective therapeutic agents for the management of pain states. In this study, we used the spinal nerve ligation (SNL) pain model to investigate the antinociceptive effect of triptolide (T10), a major active component of the traditional Chinese herb *Tripterygium wilfordii* Hook F. Intrathecal T10 inhibited the mechanical nociceptive response induced by SNL without interfering with motor performance. Additionally, the anti-nociceptive effect of T10 was associated with the inhibition of the activation of spinal astrocytes. Furthermore, intrathecal administration of T10 attenuated SNL-induced janus kinase (JAK) signal transducers and activators of transcription 3 (STAT3) signalling pathway activation and inhibited the upregulation of proinflammatory cytokines, such as interleukin-6, interleukin-1 beta, and tumour necrosis factor-**α**, in dorsal horn astrocytes. Moreover, NR2B-containing spinal N-methyl D-aspartate receptor (NMDAR) was subsequently inhibited. Above all, T10 can alleviate SNL-induced NP via inhibiting the neuroinflammation in the spinal dorsal horn. The anti-inflammation effect of T10 may be related with the suppression of spinal astrocytic JAK-STAT3 activation. Our results suggest that T10 may be a promising drug for the treatment of NP.

## 1. Introduction

Neuropathic pain (NP) causes many distressing experiences in patients. NP can be caused by damage to the peripheral or central nervous system (CNS) due to traumatic injury, surgical intervention, diabetes, and infection [1–3]. Although many drugs, such as tricyclic antidepressants and calcium channel *α*_2_-**δ** ligands, have been used to treat NP, none have achieved satisfactory pain relief, and the side effects are common [2, 4].

In recent years, substantial evidence has established that glial activation is required and sufficient for chronic pain sensitisation [5–8]. Previous studies have verified that spinal glia should be considered when treating NP. After activation, glia can release potent neuromodulators, such as proinflammatory cytokines and chemokines, as well as growth factors, which have been proven to aggravate pain hypersensitivity by enhancing neuronal excitability in the spinal dorsal horn (SDH) [6, 9, 10]. The results of our previous studies have demonstrated that the activation of microglia and astrocytes in the SDH is critical in the induction and maintenance of NP [3, 11, 12]. Specifically, astrocytes play a vital role in the maintenance of NP; the astrocyte-specific cytotoxin L-*α*-aminoadipate (LAA) can effectively relieve late-phase mechanical allodynia by interrupting the “cross-talk” between neurons and astrocytes [3, 13, 14]. In addition, accumulating evidence showed that the activation of astrocytes depends on the phosphorylation state of the janus kinase (JAK) signal transducers and activators of transcription 3 (STAT3) signalling pathway [15, 16]. Thus, the inhibition of JAK-STAT3 pathway during the activation of spinal astrocytes could be a valid approach to reduce both the peripheral nerve injury-induced hyperexcitability of dorsal horn neurons and pain responses [15, 17, 18]. The suppressor of cytokine signalling 3 (SOCS3), a physiologically inhibitory protein of the JAK-STAT3 pathway, exerts a feedback regulation of the JAK-STAT3 pathway and NP [17, 19]. 

*Tripterygium wilfordii Hook F. *(TWHF) is a traditional Chinese herb. Triptolide (T10) is one of the major active components of tripterygium extracts and has been found to have potent anti-inflammatory and immunosuppressive properties [20, 21]. TWHF has a long history of use in the treatment of rheumatoid arthritis and other autoimmune diseases [22, 23]. Recent studies have also elucidated the anti-inflammatory effects of T10 in the central nervous system (CNS) through the downregulation of the activation of astrocytes [21, 24, 25]. In a rat model of Parkinson's disease, T10 has been demonstrated to exert neuroprotective effects by suppressing inflammation in the substantia nigra [20, 21]. More importantly, it has been reported that T10 can prevent astrocytes from reactive activation by specifically blocking the JAK2-STAT3 pathway in a rat model of spinal cord injury [26]. However, reports on the mechanisms and effects of T10 on NP are scarce. In this study, we hypothesised that T10 might help to relieve NP by inhibiting the activation of astrocytes. 

In the present study, the analgesic effect of intrathecally administered T10 was explored using behavioural testing in a rat model of NP following lumbar 5 spinal nerve ligation (SNL), and the inhibition of neuroinflammation and spinal astrocytic JAK-STAT3 pathway by T10 treatment was also explored.

## 2. Methods

### 2.1. Animals

Male Sprague-Dawley rats (220–250 g) were housed in a temperature-controlled room in plastic cages (6 animals per cage) with free access to food and water at 22–25°C on a 12-hour light/dark cycle. All of the experiments reported in this study were conducted according to an experimental protocol of the Animal Use and Care Committee for Research and Education of the Fourth Military Medical University (Xi'an, China). All efforts were made to minimise the animals' suffering and the number of animals used [27].

### 2.2. Intrathecal Implantation

Intrathecal implantation was performed as described in our previous studies [13, 28, 29] by inserting polyethylene (PE) tubing through which the drug was directly injected into the subarachnoid space of the lumbar enlargement. After surgery, neurologically normal rats were injected with 2% lidocaine (10 **μ**L) through the intrathecal catheter to confirm that the PE tubing was in the subarachnoid space. Only those rats showing complete paralysis of both hind limbs and the tail after the administration of lidocaine were used for the subsequent experiments. At the end of each experiment, the position of the PE tubing in the intrathecal space at the lumbar enlargement was visually verified by exposing the lumbar spinal cord. Data from rats with incorrect PE tubing position were discarded from the study.

### 2.3. Spinal Nerve Ligation

SNL was performed according to our previous protocols [13, 28]. Briefly, rats were anesthetised with chloral hydrate (300 mg/kg, i.p.). A midline incision was then made at the L3–S2 level, and the dorsal vertebral column from L4 to S2 was exposed. After a portion of the L6 transverse process was carefully removed, the left L5 spinal nerve was carefully isolated and tightly ligated distal to the dorsal root ganglion (DRG) with 6-0 silk thread. Sham-operated animals were subjected to a similar surgical procedure in which the isolated spinal nerves were not ligated.

### 2.4. Drug Administration

T10, obtained from Fujian Academy of Medical Sciences (Fujian, China), was purified from TWHF. The purity of the T10 was greater than 99%. T10 was dissolved in DMSO and was then diluted with 0.9% (w/v) saline solution. The doses for the intrathecal administration of T10 were selected according to a previous study and our preliminary experiments [30]. Animals were divided into 4 groups for administration: a Sham-Vehicle group (*n* = 16, a volume of 10 *μ*L normal saline was injected into Sham-operated rats), an SNL-Vehicle group (*n* = 16, a volume of 10 *μ*L normal saline was injected into SNL rats), an SNL-T10 group (*n* = 48, 16 for each of the 3 subgroups; 10 *μ*L of T10 (3, 10, or 30 **μ**g/kg for each subgroup) was injected into SNL rats, resp.), and a Sham-T10 group (*n* = 16, a volume of 10 *μ*L of T10 (30 **μ**g/kg) was injected into Sham-operated rats).

### 2.5. Rotarod Testing

Motor dysfunction can have evident effects on nociceptive behavioural tests [31]. To assess whether the intrathecal administration of T10 influenced motor function, rotarod tests were performed on rats using an accelerating rotating rod (Ugo Basile 7650, Varese, Italy). After 1 min of training, rats were placed on the rotarod, which was linearly accelerated from 4 to 40 rpm over 5 min. The elapsed time before the rat fell on each of three runs with 10 min intervals between runs was recorded for each rat. The test was repeated 30 min after the intrathecal administration of T10 or saline once per day for 7 days, and the time that the rat remained on the rotarod was recorded. Final results are expressed as a percentage of each rat's baseline value.

### 2.6. Mechanical Hypersensitivity

Rats were placed on an elevated mesh grid that completely exposed the middle of the hind paw. Mechanical hypersensitivity was tested using von Frey filaments (Stoelting, Kiel, WI, USA) by experimenters who were blinded to group assignment [32]. The stiffness of the von Frey filaments was 2, 4, 6, 8, 10, 15, and 26 g. The hind paw was pressed with each filament, in the order of increasing stiffness, for 5 s. Rapid pulling back, biting, or shaking the hind limb within 5 s of the hind limb being pricked by one of the von Frey filaments was taken as a positive sign of withdrawal. The interval between trials was at least 5 min. For each trial, the same hind limb was stimulated 10 times by a single von Frey filament before being stimulated by the next larger filament. The minimal value that resulted in at least six responses to 10 stimulations was recorded. The formula for calculating the percentage change was 100 − 100 × (baseline of SNL-T10−post-SNL-T10)/(baseline of SNL-Veh − post-SNL-Veh).

### 2.7. Immunofluorescent Histochemistry

The rats were deeply anesthetised by injection of pentobarbital (60 mg/kg, i.p.) and transcardially perfused with 200 mL of 5 mM sodium phosphate-buffered 0.9% (w/v) saline (PBS, pH 7.3), followed by 500 mL of 4% (w/v) paraformaldehyde in 0.1 M phosphate buffer (PB, pH 7.4). The L5 spinal cord segments were harvested and immersed in 30% (w/v) sucrose in 0.1 M PB overnight at 4°C. Transverse spinal sections (25 **μ**m thickness) were then cut on a cryostat (Leica CM1800; Heidelberg, Germany). The sections were rinsed in PBS (pH 7.2–7.4) 3 times (10 min each) and blocked for 1 hour at 37°C in 0.01 M PBS containing 10% normal goat serum and 0.3% Triton X-100. The sections were incubated for 2 h at room temperature and then for 48 h at 4°C with a mixture of antiglial fibrillary acidic protein (GFAP) mouse IgG (1 : 5000; Chemicon, Temecula, CA, USA) and anti-pSTAT3 rabbit IgG (1 : 1000; Cell Signalling, MA, USA) in PBS containing 0.3% (v/v) Triton X-100, 0.25% (w/v) **λ**-carrageenan, and 5% (v/v) donkey serum (PBS-XCD). After being washed three times in 0.01 M PBS (10 min each), the sections were incubated for 4 h at room temperature with a mixture of 10 *μ*g/mL Alexa488-conjugated donkey antibody to rabbit IgG and 10 *μ*g/mL Alexa594-conjugated donkey antibody to mouse IgG (1 : 500; Millipore, Billerica, MA, USA). After staining, all of the sections were mounted onto glass slides and cover-slipped with 50% (v/v) glycerol and 2.5% (w/v) triethylenediamine (antifading agent) in 0.05 M PBS. Using a confocal laser-scanning microscope (FV1000; Olympus, Tokyo, Japan), the sections were observed with the appropriate laser beams and filter settings for Alexa488 (excitation, 488 nm; emission, 510–530 nm) and Alexa594 (excitation, 543 nm; emission, 590–615 nm). The digital images were captured using FV10-ASW-1.6 software (Olympus), modified (15–20% contrast enhancement) in Photoshop CS2 (Adobe Systems, San Jose, CA) and then saved as TIFF files.

### 2.8. Western Blot Analysis

Animals were deeply anesthetised by injection of pentobarbital (60 mg/kg, i.p.) and then rapidly sacrificed. The L5 spinal cord segments were dissected on ice according to the termination of the L4 and L5 dorsal roots. The left dorsal part of spinal cord was further split and then homogenised with a hand-held pestle in SDS sample buffer (10 mL/mg tissue) containing a mixture of proteinase and phosphatase inhibitors (Sigma, MO, USA). The protein concentrations were estimated using the bicinchoninic acid (BCA) method. The samples were heated in boiling water for 8 min, loaded onto 10% SDS-polyacrylamide gels (Bio-Rad Laboratories, CA, USA), and transferred to polyvinylidene difluoride membranes (PVDF, Immobilon-P, Millipore, Billerica, MA, USA). Membranes were blocked in a 5% BSA solution for 2 hours and probed with the following primary antibodies overnight at 4°C: anti-GFAP mouse IgG (1 : 5000; Chemicon, Temecula, CA, USA), anti-pSTAT3 rabbit IgG (1 : 300; Cell Signalling, MA, USA), anti-pJAK2 rabbit IgG (1 : 300; Cell Signalling, MA, USA), anti-pNR2B rabbit IgG (1 : 100; Santa-Cruz Biotechnology, Santa Cruz, California, USA), and anti-**β**-actin mouse IgG (1 : 3000, Sigma). The membranes were then incubated with the following secondary antibodies for 2 hours: HRP-coagulated anti-rabbit donkey IgG (1 : 5000; Zhongshan, Beijing, China) and HRP-coagulated anti-mouse donkey IgG (1 : 5000; Zhongshan). The membranes were rinsed three times (10 minutes each) with Tris-buffered saline with Tween-20 (TBST) between each step. All reactions were detected by the enhanced chemiluminescence (ECL) detection method (Amersham). The densities of protein blots were analyzed using Labworks Software (Ultra-Violet Products, UK). The densities of target proteins and *β*-actin immunoreactive bands were quantified with background subtraction. The same size of square was drawn around each band to measure the density and the background near that band was subtracted. Target protein levels were normalized against *β*-actin levels and expressed as relative fold changes compared to the Sham-Veh group. The intensity of blots was quantified with densitometry by the persons who were blind to the different treatments.

### 2.9. Enzyme-Linked Immunosorbent Assay

The left dorsal horns of the L5 spinal segments of animals in different groups were split according to the same method used for western blot analysis. The amounts of IL-1**β**, IL-6, and TNF-**α** were measured by enzyme-linked immunosorbent assays using the Multi-Analyte ELISArray Kit System (Mix-N-Match; SABiosciences, Frederick, MD, USA) according to the manufacturer's instructions.

### 2.10. Real-Time Quantitative Polymerase Chain Reactions

Rats were deeply anesthetised with pentobarbital (60 mg/kg) and sacrificed. Total RNA from the L5 spinal segments was extracted with Trizol (GIBCO/BRL Life Technologies Inc., Grand Island, NY, USA). Complementary DNA (cDNA) was synthesised with oligo (dT) 12–18 using Superscript III Reverse Transcriptase for RT-PCR (Invitrogen, Carlsbad, CA, USA). The designs of the primers used in the present study are shown in Table 1. Equal amounts of RNA (1 **μ**g) were used to prepare cDNA using the SYBR Premix Ex Taq TM (Takara, Tokyo, Japan) and analysed with a real-time PCR detection system (Applied Biosystems, Foster City, CA, USA). The data were analysed with 7500 System SDS Software 1.3.1 (Applied Biosystems) using the standard curve method. All values were normalised to GAPDH expression.

### 2.11. Quantification and Statistical Analyses

#### 2.11.1. Statistical Analyses

All data were collected and analysed by researchers blinded to the surgery and reagents that were used. Repeated measures ANOVA (with Bonferroni confidence interval adjustment) tests were conducted for the data from the rotarod and vonFrey experiments. Data from the western blot, real-time PCR, and ELISA tests were analysed using a one-way ANOVA followed by the Newman–Keuls test for post hoc analysis. Pearson correlation analysis was used to identify correlations between the analgesic effect of T10 and the expressions of the related proteins. All of the data are presented as mean ± SEM, and all statistical analyses were performed using SPSS software version 16.0 (SPSS Inc., Chicago, IL, USA). A *P* value of <0.05 was considered statistically significant.

#### 2.11.2. Dose-Effect Curve and ED_50_ Calculation

The T10 dosages were transformed into logarithm dose with Prism and the nonline fit was performed so as to build the dose-effect curve. Based on the dose-effect curve, the ED_50_ of the effects of T10 on analgesia was calculated. The reliability of the ED_50_ calculated from a specific dose-effect curve was evaluated using the slope factor generated by the GraphPad Prism version 5.01 for Windows (San Diego, CA, USA, http://www.graphpad.com/).

## 3. Results

### 3.1. Effects of the Intrathecal Administration of T10 on Motor Function in Rotarod Testing

**Figure 1:**
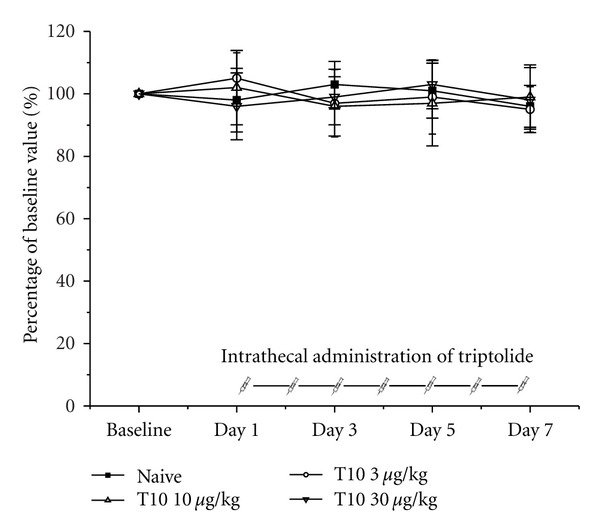
The effects of intrathecal triptolide on motor performance in the rotarod test in normal rats. The intrathecal administration of triptolide (3, 10, or 30 *μ*g/kg) did not have an effect on motor performance relative to the baseline response. The results are expressed as a percentage of the baseline values. Each group consisted of six rats.

Motor dysfunction can produce evident effects in nociceptive behavioural tests. Thus, it was essential to assess whether the dosages of T10 administered in the present study (3, 10 and 30 **μ**g/kg) impaired motor functions. Another 24 rats were used to assess the influence of T10 administration on motor function as measured by the rotarod test. The intrathecal administration of T10 (3, 10, and 30 **μ**g/kg) did not have obvious effects on the motor function of rats 30 min after injection compared to their own baselines (Figure 1).

### 3.2. Intrathecal Administration of T10 Attenuates SNL-Induced Mechanical Allodynia in a Dose-Dependent Manner

**Figure 2:**
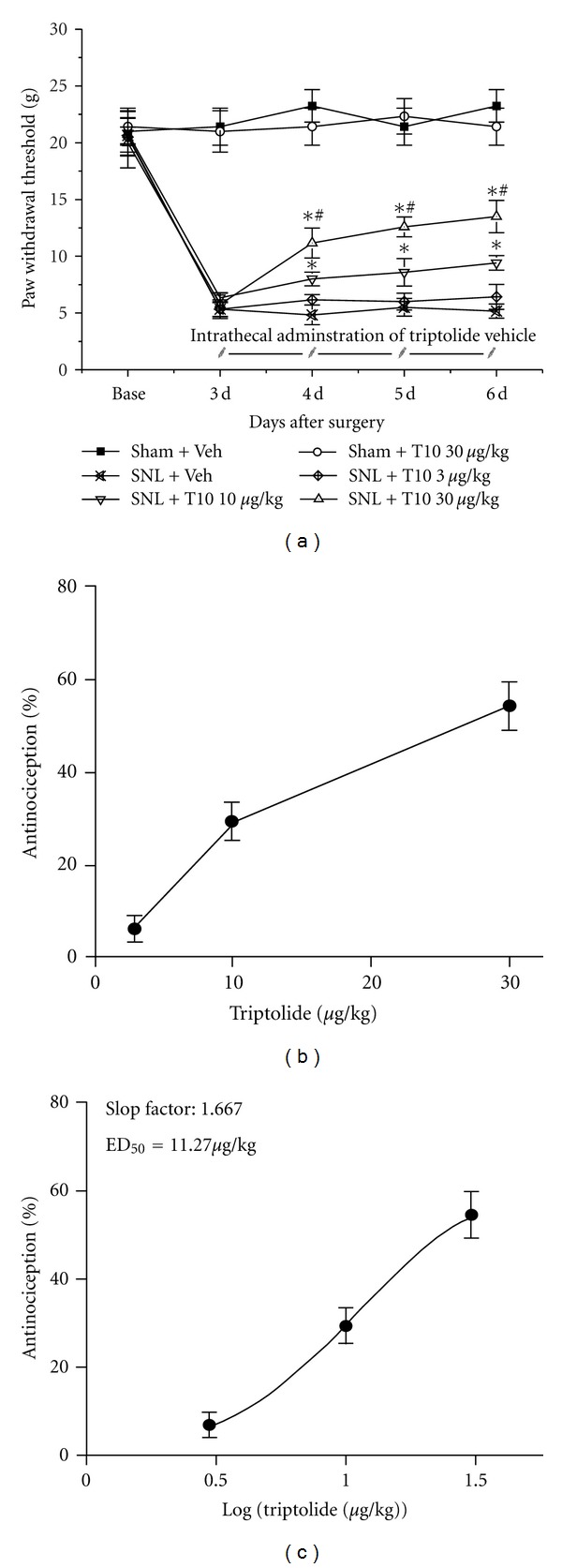
The effects of intrathecal triptolide on SNL-induced mechanical allodynia. SNL injury resulted in prominent mechanical allodynia. However, the intrathecal injection of triptolide did not affect the pain threshold of the Sham-operated group. The intrathecal administration of triptolide (10 or 30 **μ**g/kg, once per day from POD 3 to 6) significantly blocked SNL-induced mechanical allodynia in a dose-dependent manner; in contrast, 3 *μ*g/kg/d of intrathecal triptolide did not alter the SNL-induced mechanical allodynia (a). SNL: spinal nerve ligation; POD: postoperative day. *P  < 0.05, compared to the SNL-Veh group. ^#^ *P *<0.05, compared to the SNL-T10 10 *μ*g/kg group. The dose-effect and log- (dose-) effect curves for analgesic effects of triptolide are shown in (b) and (c). Each group consisted of 16 rats.

We assessed whether T10 prevented nociception in the neuropathic pain model of SNL. Different doses (3, 10, and 30 **μ**g/kg) of T10 were injected once per day from postoperative day (POD) 3 to 6, and behavioural experiments were performed 30 min after each injection (Figure 2(a)). The SNL injury resulted in prominent mechanical allodynia as shown in the SNL-Veh group (*P* < 0.05). Compared with the SNL-Veh group, a single intrathecal administration of T10 produced a significant and dose-dependent reduction of SNL-induced mechanical hyperalgesia in the paw ipsilateral to the SNL, as shown in Figure 2(a). However, neither the high (30 **μ**g/kg; Figure 2(a)) nor the low (3 and 10 **μ**g/kg, data not shown) doses of T10 altered the basal threshold in the Sham-operated group. The effect of T10 on SNL-induced mechanical allodynia was calculated based on the log- (dose-) response curve (Figure 2(b)) that was calculated from the dose-response curve (Figure 2(c)). The ED_50_ of T10 on SNL-induced mechanical allodynia was 11.27 **μ**g/kg, and the slope factor was 1.667, suggesting that our regimen for dosage selection was robust. Thus, the dose of 10 **μ**g/kg, which was similar to the ED_50_ of T10 for SNL-induced mechanical allodynia, was chosen for subsequent experiments.

### 3.3. The Effects of Intrathecal T10 on SNL-Induced Elevated GFAP Expression

**Figure 3:**
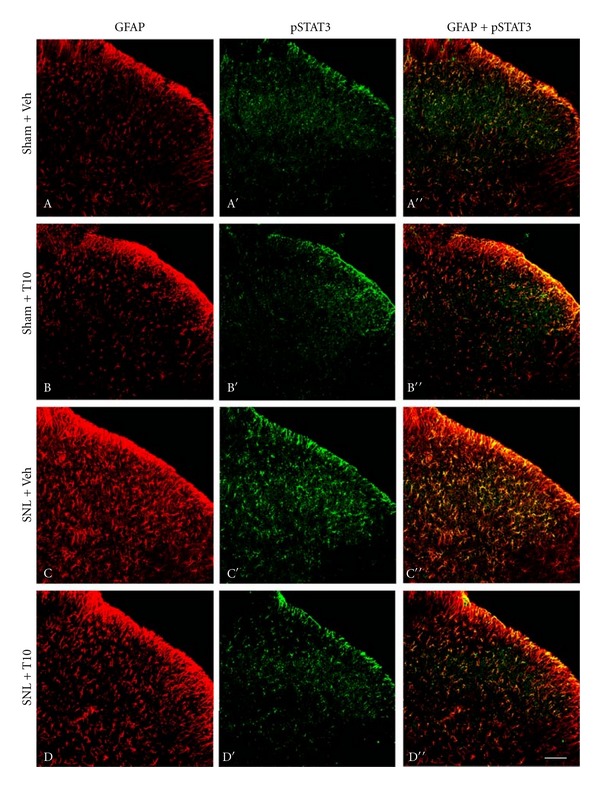
The effects of intrathecal triptolide (10 *μ*g/kg) on SNL-induced GFAP and pSTAT3 in the SDH of the rat by double-immunofluorescence microphotographs on POD 6. The Sham surgery induced weak p-STAT3 and GFAP-immunoreactivity (-IR) in the ipsilateral dorsal spinal cord (A, A′, and A′′). Changes in GFAP-IR and p-STAT3-IR were not observed in the Sham group after intrathecal triptolide application (B, B′, and B′′). After SNL surgery, increased GFAP-IR and p-STAT3-IR were detected ipsilateral to the lesion of the SDH, and many of the p-STAT3-IRs were colabelled with GFAP-IR, indicating that p-STAT3 accumulated in spinal cord astrocytes on POD 6 (C, C′, and C′′). The intrathecal administration of triptolide inhibited the expression of GFAP- and p-STAT3-IR in the ipsilateral SDH after SNL (D, D′, and D′′). SNL, spinal nerve ligation; POD, postoperative day, SDH, spinal dorsal horn. Scale bar, 100 **μ**m.

**Figure 4:**
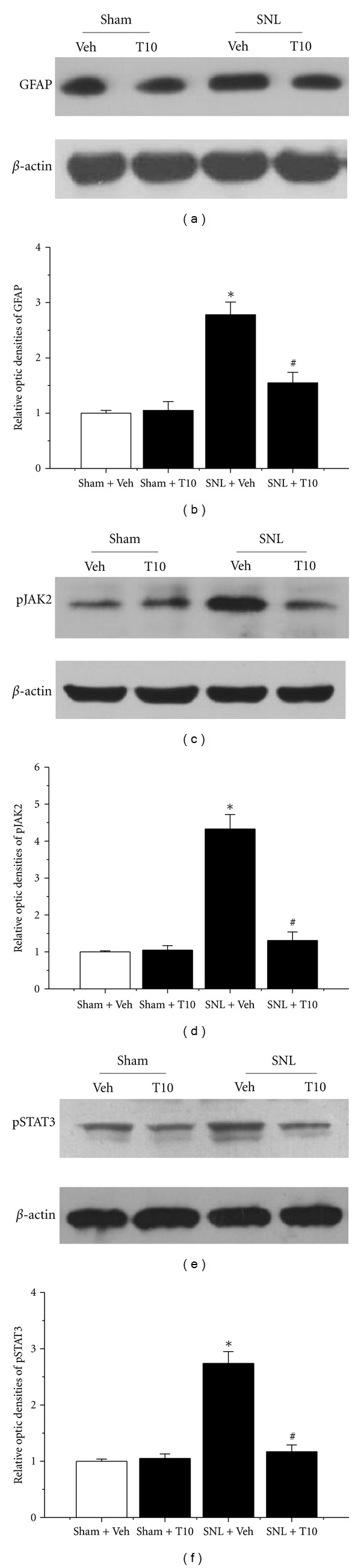
Western blot quantification of the expression of GFAP, pJAK2, and pSTAT3 after intrathecal administration of triptolide. (a) SNL injury resulted in increased GFAP expression at POD 6 in the ipsilateral SDH. SNL-induced elevations in GFAP expression were completely inhibited in rats intrathecally injected with triptolide (10 **μ**g/kg). (b) The levels of the phosphorylated forms of JAK2 (pJAK2) and STAT3 (pSTAT3) were increased in the ipsilateral SDH of SNL rats at POD 6. The intrathecal administration of triptolide (10 **μ**g/kg) prevented SNL-induced pJAK2 and pSTAT3 accumulation. SNL, spinal nerve ligation; SDH, spinal dorsal horn; POD, postoperative day. *P < 0.05, SNL-Veh group versus Sham-Veh group; ^#^ *P* < 0.05, SNL-T10 group versus SNL-Veh group. The results are expressed as relative fold changes compared to the Sham-Veh group. Each group consisted of 12 rats.

We investigated GFAP expression on POD 3 and POD 6 in various groups to identify whether the antiallodynic and antihyperalgesic effects of T10 were accompanied by the inhibition of astrocytic activation (Figures 3 and 4). Immunohistochemical data showed SNL-induced enhancement of GFAP expression in the ipsilateral SDH on POD 6 (Figures 3(B) and 3(C)). Additionally, the astrocytes activated by SNL had hypertrophied cell bodies and thickened processes. Enhanced GFAP expression induced by SNL was also verified by western blot analysis (*P* < 0.05, compared to that of Sham-Veh; Figures 4(a) and 4(b)). In contrast, the intrathecal administration of T10 from POD 3 to POD 6 resulted in decreased GFAP expression in the dorsal horn by western blot analysis (*P* < 0.05, compared to that of SNL-Veh; Figures 4(a) and 4(b)). Immunohistochemical data also showed the astrocytes of the SNL-T10 group were not in an activated state and were similar to those of the rats in a naïve state (Figure 3(D)).

### 3.4. The Effects of Intrathecal T10 on SNL-Induced JAK2-STAT3 Pathway Activation

**Figure 5:**
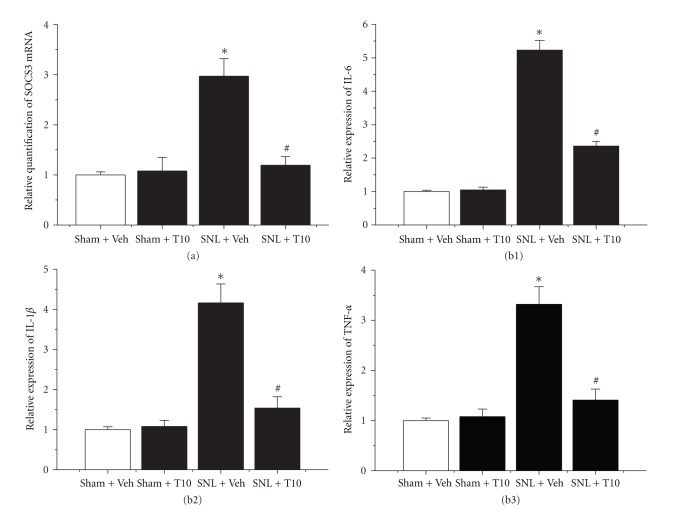
The effects of intrathecal triptolide on the STAT3 target gene SOCS3 (a) and markers associated with spinal cord inflammatory state ((b1)–(b3)). (a) SOCS3 mRNA levels in rats that received different treatments were determined in the ipsilateral L4–L5 segment of the SDH using semiquantitative RT-PCR. SNL injury resulted in increased SOCS3 mRNA expression at 6 d after surgery in the ipsilateral SDH. This effect was prevented by treatment with triptolide (10 **μ**g/kg). (b) The protein levels of the proinflammatory cytokines interleukin 6 (b1), interleukin-1 **β** (b2), and tumour necrosis factor-**α** (b3) were determined in the ipsilateral L4–L5 segment of the dorsal spinal cord in rats that received different treatments using enzyme-linked immunosorbent assay. These cytokines were increased at POD 6 in the ipsilateral SDH. SNL-induced increases of proinflammatory cytokine expression were prevented by the treatment with triptolide (10 **μ**g/kg). SNL, spinal nerve ligation; SDH, spinal dorsal horn; POD, postoperative day. *P < 0.05, SNL-Veh group versus Sham-Veh group; ^#^ *P* < 0.05, SNL-T10 group versus SNL-Veh group. The levels of SOCS3 mRNA and proinflammatory cytokines are expressed as relative fold changes compared to the Sham-Veh group. Each group consisted of four rats.

Additionally, the effect of intrathecal administration of T10 on STAT3 phosphorylation was tested. Enhanced pSTAT3-immunoreactivity was observed in the ipsilateral SDH on POD 6 in SNL rats, whereas weak pSTAT3-immunoreactivity was observed in naïve rats. In addition, almost all pSTAT3-positive cells were double-labelled with GFAP (Figures 3(C) and 3(C′′)). To further examine whether the JAK2-STAT3 pathway was involved in the suppressive effect of T10 on SNL-induced astrocytic activation, we assessed the levels of phosphorylation of JAK2 and STAT3 using western blot analysis. Similar to the effect of T10 on GFAP expression, the SNL-induced elevation of the levels of pSTAT3 was also significantly attenuated (*P* < 0.05, compared to that of SNL-Veh; Figures 4(e) and 4(f)). A similar result was obtained for pJAK2 expression. The amount of pJAK2 was also inhibited by T10 (*P* < 0.05, compared to that of SNL-Veh; Figures 4(c) and 4(d)). To further evaluate the effect of T10 on the JAK-STAT3 pathway, the expression profile of the STAT3 target gene SOCS3 was determined. Real-time PCR showed that the markedly enhanced levels of SOCS3 mRNA were associated with peripheral nerve injury. Additionally, the administration of T10 also decreased the SNL-induced increase of the SOCS3 mRNA concentration (*P* < 0.05, compared to that of SNL-Veh; Figure 5(a)).

### 3.5. The Effects of Intrathecal T10 on SNL-Induced Elevated Proinflammatory Cytokine Expression

Proinflammatory cytokine (TNF-*α*, IL-1*β*, and IL-6) expression was analysed using enzyme-linked immunosorbent assays. The administration of T10 did not change spinal proinflammatory cytokine expression of normal rats (Figures 5(b1), 5(b2), and 5(b3). However, SNL resulted in significantly increased proinflammatory cytokine expression in the ipsilateral SDH on POD 6 (*P* < 0.05, compared to Sham-Veh; Figures 5(b1), 5(b2), and 5(b3). Additionally, there was a significant downregulation of proinflammatory cytokines in the T10 administration group that was subjected to SNL (*P* < 0.05, compared to SNL-Veh; Figures 5(b1), 5(b2), and 5(b3).

### 3.6. The Effects of Intrathecal T10 on SNL-Induced Activation of NR2B-Containing Spinal N-Methyl D-Aspartate Receptor (NMDAR)

**Figure 6:**
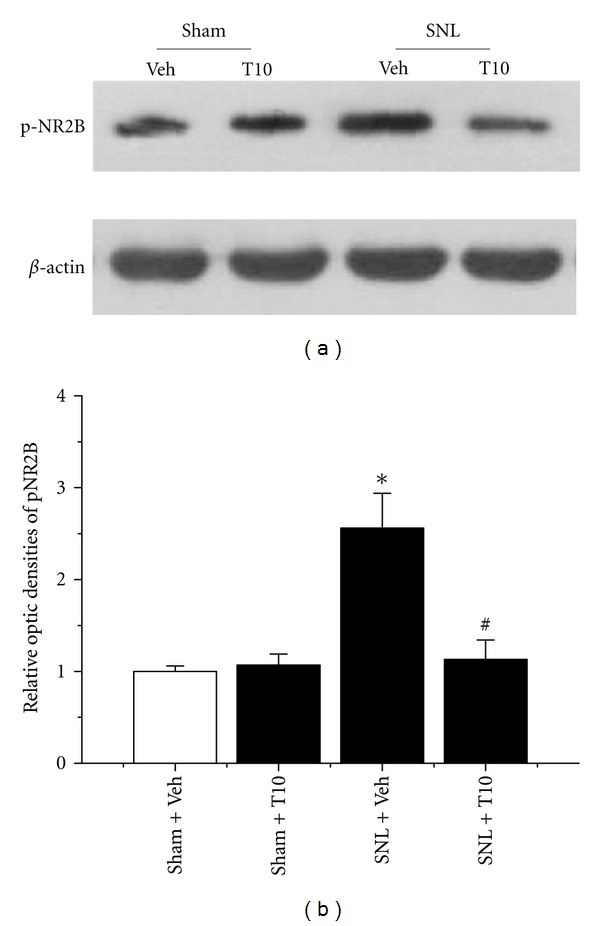
The effects of intrathecal triptolide on the expression of phosphorylated forms of NR2B (pNR2B). SNL injury resulted in increased levels of pNR2B in the ipsilateral SDH at POD 6. The intrathecal administration of triptolide (10 **μ**g/kg) also prevented SNL-evoked pNR2B accumulation. SNL, spinal nerve ligation; SDH, spinal dorsal horn; POD, postoperative day. *P < 0.05, SNL-Veh group versus Sham-Veh group; ^#^ *P* < 0.05, SNL-T10 group versus SNL-Veh group. The levels of pNR2B are expressed as relative fold changes compared to the Sham-Veh group. Each group consisted of 12 rats.

We also determined whether the intrathecal administration of T10 affected the SNL-induced activation of NMDARs, which is demonstrated via phosphorylation. The expression of pNR2B in each group was detected by western blot analysis (Figure 6). We found a significant increase in pNR2B in the SDH ipsilateral to the SNL (*P* < 0.05 compared to Sham-Veh; Figures 6(a) and 6(b)). However, the SNL-induced increase of pNR2B was markedly suppressed by treatment with T10 (*P* < 0.05, compared to SNL-Veh; Figures 6(a) and 6(b)).

### 3.7. The Antinociceptive Properties of T10 Are Associated with the Expression of Related Proteins

**Figure 7:**
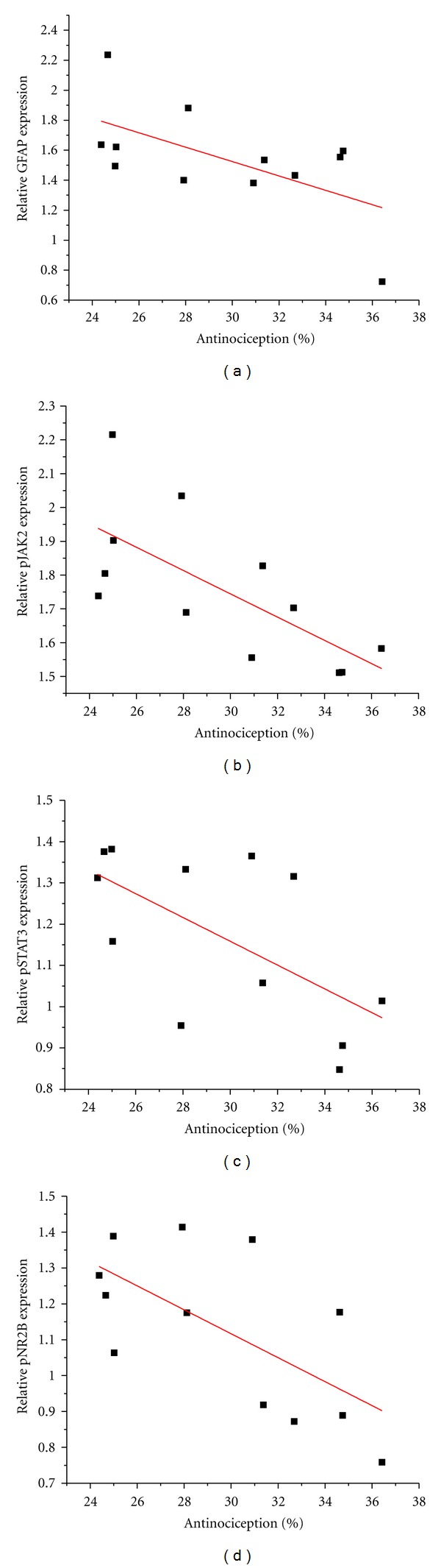
The regulation of the expression of related proteins by triptolide after SNL (10 *μ*g/kg) was negatively correlated with the analgesic effect of triptolide. GFAP (*r* = −0.601; *P* = 0.039), pJAK2 (*r* = −0.697; *P* = 0.012), pSTAT3 (*r* = −0.625; *P* = 0.030), and pNR2B (*r* = −0.651; *P* = 0.022) were associated with the analgesic effect of triptolide (a–d). SNL, spinal nerve ligation. Each group consisted of twelve rats. *r*, Pearson correlation coefficient; *P*, statistical significance of *r*. Each group consisted of 12 rats.

The relationships between the analgesic effect of T10 and the expressions of GFAP, pJAK2, pSTAT3, and pNR2B, as revealed by linear regression analyses, are shown in Figure 7. The Pearson correlation coefficient between the analgesic effect of T10 and the expression of GFAP was −0.601 (*P* = 0.039; Figure 7(a)), and the Pearson correlation coefficients between the analgesic effect of T10 and the expression of pJAK2 and pSTAT3 were −0.697 (*P* = 0.012; Figure 7(b)) and −0.625 (*P* = 0.030; Figure 7(c)), respectively. Finally, the Pearson correlation coefficient between the analgesic effect of T10 and the expression of pNR2B was −0.651 (*P* = 0.022; Figure 7(d)).

## 4. Discussion

In the present study, we observed that intrathecal T10 significantly attenuates the mechanical allodynia induced by SNL at doses of 10 and 30 **μ**g/kg. Subsequently, we investigated the mechanisms that underlie the antiallodynic effects of T10. We found that intrathecal T10 (10 **μ**g/kg) inhibits the proliferation of reactive astrocytes in the lumbar SDH. Interestingly, the SNL-induced activation of the JAK-STAT3 pathway was inhibited by T10, which is important for regulating astrocytic activation. Additionally, T10 treatment also resulted in lower levels of proinflammatory cytokines in the SDH, especially IL-6. Together, our findings suggest that the JAK-STAT3 pathway suppression is involved in the analgesic effect of T10.

The present study showed that the intrathecal administration of T10 inhibited SNL-induced mechanical allodynia (as indicated by the vonFrey test) in a dose-dependent manner. A growing body of evidence indicates that reactive astrocytes, in addition to microglia, are crucial for the maintenance of neuropathic pain [3, 33]. Once activated, glial cells release inflammatory stimulants including cytokines, prostaglandins and neurotrophic factors that can change the polarisation characteristics of the afferent neurons and thus modulate the transmission of pain information in the CNS [34–36]. Based on the ED_50_ of T10 for SNL-induced mechanical allodynia, a dose of 10 **μ**g/kg was chosen to explore the mechanism of the antiallodynic effects of T10. Our results showed that T10 downregulated GFAP expression in the SDH after SNL injury at the dose of 10 **μ**g/kg. T10 has been reported to play important roles in modulating immune function both within and outside of the nervous system [20, 24, 37–39]. It has been reported that T10 could be useful in the treatment of a variety of inflammatory and autoimmune diseases, such as rheumatoid arthritis [23], and increasing evidence shows that T10 can inhibit glia activation in rat models of Parkinson's [20, 38] and Alzheimer's diseases [39] by inhibiting the release of proinflammatory and cytotoxic factors, such as TNF-*α* and IL-1*β*. The inhibitory effects of T10 on neuroinflammation are in agreement with several other studies regarding the effects of T10 on other diseases. Additionally, our correlation analysis showed that the regulation of GFAP expression after SNL by T10 had a negative correlation with the analgesic effect of T10. Based on these findings, the inhibition of spinal astrocytic activation may be involved in the analgesic effect of T10. 

The JAK-STAT pathway is a vital signalling mechanism for responding to various extracellular stimuli; this pathway transduces signals from the cell surface to the nucleus that are associated with cell proliferation, differentiation, cell migration, and apoptosis [16, 40, 41]. The JAK-STAT pathway plays an important role in the immune/inflammatory response. Several studies have implicated JAK-STAT3 signalling in astrogliosis under many neuropathological conditions, such as spinal cord injury [16, 42], brain ischemia [43, 44], and neuropathic pain [15, 18]. Recent evidence indicates that the astrocytic JAK/STAT3 pathway is crucial for regulating the astrocyte proliferation that participates in the maintenance of peripheral nerve injury-induced allodynia from 2–6 days after nerve injury in the SDH of rats [15]. However, multiple conflicting studies have reported that increases in phosphorylated STAT3 are only involved in the activation of microglia after SNL injury in rats [17, 18]. This discrepancy is attributed to the varied time windows that were analysed after SNL injury. Another study using a spinal cord injury model reported that JAK-STAT3 activation in various cell types is dependent on the time after injury [42]. Our results verified that astrocytic JAK2-STAT3 signalling is activated in the window between days 5 and 7 after SNL. Additionally, our present study showed that the administration of T10 throughout this time window attenuated pJAK2 and pSTAT3 expression and was accompanied by suppressed astrocytic activation. In contrast, decreases in STAT3 nuclear translocation in astrocytes were observed in the T10 treatment group. The correlation analysis also showed that the expressions of the pJAK2 and pSTAT3 after SNL by T10 were negatively correlated with the analgesic effect of T10. Furthermore, T10 can suppress the expression of SOCS3 mRNA, which is a negative feedback inhibitor of the JAK-STAT3 pathway. In response to peripheral nerve injury, increased SOCS3 protein expression can block the JAK-STAT3 pathway and its downstream signalling. Thus, all of our findings suggest that the inhibition of spinal astrocytic JAK2-STAT3 pathway activation may be involved in the analgesic effects of T10.

Increased levels of TNF-*α*, IL-1*β*, and IL-6 have been observed both in the DRG and SDH in the spinal nerve ligation model of neuropathic pain [10, 45–47]. A growing body of recent evidence supports an important role for proinflammatory cytokines in pain sensitisation during the development of NP [45, 46, 48]. It has been reported that IL-1*β* and TNF-*α* are involved in the initiation of NP, whereas IL-6 is important for the maintenance of NP [45, 49, 50]. Additionally, the increased expression of IL-6 in the SDH is directly induced by IL-1*β* and TNF-*α* [36, 50]. As an immunosuppressant, T10 can inhibit proinflammatory cytokine production in primary cultured microglia and protect dopaminergic neurons from the damage induced by inflammation [38]. Moreover, IL-6 is the predominant activator of the JAK-STAT3 pathway [15, 17, 51]. Our present results showed that intrathecal T10 can downregulate the levels of TNF-*α*, IL-1*β* and especially IL-6 in the SDH on POD 6 after SNL. These results are also consistent with JAK-STAT3 inhibition. 

The NMDAR in the superficial SDH has been implicated as a major contributor to excitatory nociceptive transmission. An increase in glutamate NMDA receptor-mediated excitatory synaptic transmission in the SDH neurons can manifest as nerve injury-induced central sensitisation [28]. Substantial evidence has shown that NR2B subunit, a kind of functional NMDAR, may play a role in pain transmission and the development of chronic pain [28, 52]. It has been reported that the expression of NR2B subunit increases in SDH neurons beginning 48 h after SNL in rats. This increase reaches its peak at 3 days, lasts for 14 days, and returns to preoperation levels 28 days after SNL [52]. The activation of astrocytes leads to a consequent release of neuroexcitatory substances, including prostaglandins, IL-1, IL-6, and subsequently induces the phosphorylation of NR2B subunit in spinal neurons [10, 53, 54]. In the present study, western blot analysis revealed that intrathecal T10 can inhibit the SNL-induced elevation of pNR2B expression. Moreover, the regulation of the expression of pNR2B after SNL by T10 was negatively correlated with the analgesic effect of T10. Therefore, we speculate that the regulation of the expression of pNR2B after SNL by T10 may be correlated with the inhibition of astrocytic activation.

## 5. Conclusions

These results presented here strongly suggest that intrathecal T10 (3, 10, 30 **μ**g/kg) produces a consistent and dose-dependent antinociceptive effect in SNL-induced NP. One possible mechanism involving in the analgesic effects of T10 is that T10 downregulates the levels of TNF-**α**, IL-1**β**, and IL-6, which subsequently inhibit the astrocytic activation by the suppression of the JAK-STAT3 signalling pathway. Intrathecal T10 can also decrease NR2B-containing NMDAR phosphorylation in SDH neurons, thereby attenuating SNL-induced central sensitisation. Our results suggest that T10 may be a promising drug for the treatment of NP.

## Figures and Tables

**Table 1:** Primers sequence for the rat genes characterized in this experiment.

Genes	Primers	Sequences	Accession number
SCOCS3	Forward primer	5′CCCGCTTTGACTGTGTACT3′	NM053565
	Reverse primer	5′TGAGTACCAGCGGGATCTTCTC3′	

GAPDH	Forward primer	5′-CCCCCAATGTATCCGTTGTG-3′	NM01008
	Reverse primer	5′-TAGCCCAGGATGCCCTTTAGT-3′	
